# Nonuse and Nonpurchase of Assistive Devices After Stroke: An Exploratory Interview Study of Retrospective Device Histories Among Community-Dwelling Adults

**DOI:** 10.3390/healthcare14193327

**Published:** 2026-10-06

**Authors:** EunRae Ro, Kwang-Ok An, Sung-Yong Lee, Dong-A Kim, Seon-Deok Eun

**Affiliations:** 1 Assistive Technology Research Team for Independent Living, National Rehabilitation Center, Seoul 01022, Republic of Korea; rea0930@korea.kr (E.R.); anko04@korea.kr (K.-O.A.); lsyong1625@korea.kr (S.-Y.L.); 2 Division of Public Medical Rehabilitation, National Rehabilitation Center, Seoul 01022, Republic of Korea; 3 Department of Healthcare and Public Health Research, National Rehabilitation Center, Seoul 01022, Republic of Korea

**Keywords:** stroke, assistive technology, device discontinuation, assistive device abandonment, unmet need, rehabilitation, qualitative research

## Abstract

**Highlights:**

**What are the main findings?**

- Reported nonuse after stroke was concentrated among devices used during inpatient rehabilitation (standing frames, walkers, and manual wheelchairs); functional improvement was among the reasons recorded, but for most of these devices it co-occurred with cost, bulk, or replacement by another device.
- A separate cluster of nonuse arose from body-worn devices, chiefly ankle-foot orthoses, that could not keep pace with a continuously changing hemiplegic limb.

**What are the implications of the main findings?**

- Separating discontinuation because a device is no longer needed from abandonment because it no longer fits may clarify nonuse figures, although in retrospective joint participant–caregiver accounts the two could not always be distinguished.
- Whether ownership-based provision with fixed durability periods fits stage-limited need, and whether rental or prescription-based pathways would fit it better, are hypotheses for prospective evaluation rather than conclusions of this study.

**Abstract:**

Background/Objectives: Because function typically improves after stroke, the devices people need, and for how long, may change along the recovery trajectory. This exploratory study examined retrospective accounts of assistive device use, discontinuation, replacement, and nonpurchase across two post-onset stages, hospitalization and home and community reintegration. Methods: Ten community-dwelling adults with chronic stroke (mean disability duration 13.9 years) were interviewed twice at home, with caregivers contributing where present, and reconstructed their device histories from recall; the resulting accounts are treated as joint participant–caregiver reports. Instruments followed the Human–Activity–Assistive Technology (HAAT) model; field notes and completed device checklists were analyzed by inductive qualitative content analysis, and facilitators and barriers were classified with the World Health Organization people-centered assistive technology (5P) framework. Aggregate figures for the stroke subsample of the 2020 National Survey on Persons with Disabilities (*n* = 662) are reported as national context rather than as independent confirmation. Results: Participants reported having used a mean of 11.2 device types, yet named only 1.3 devices that were needed but not purchased. Discontinuation was concentrated among devices used during inpatient rehabilitation: standing frames (9/9), walkers (5/5), and manual wheelchairs (8/10) had been set aside. Functional improvement was the only reason recorded for walkers, but for standing frames and manual wheelchairs it was recorded alongside cost, bulk, replacement, and, in one case, continuing unmet need, so its independent contribution could not be established. A second cluster reflected the poor fit of body-worn devices to a changing hemiplegic limb (ankle-foot orthoses, 4/6). The nonpurchase barrier reported most often was inability to find a suitable product on the market. Conclusions: These retrospective accounts from ten participants cannot support policy conclusions. They generate hypotheses, requiring prospective testing, about whether ownership-based funding with fixed durability periods fits stage-limited need and whether rental provision, prescription-based provision for orthoses, and targeted product development would fit it better.

## 1. Introduction

### 1.1. Background and Rationale

Stroke is one of the leading causes of acquired disability in adults. In South Korea, approximately 105,000 people are estimated to experience a new stroke each year [1], and stroke accounts for a substantial share of the brain lesion disability population registered under the national disability system [2]. People with stroke present complex and heterogeneous impairments that extend across motor and movement-related functions, sensory functions and pain, and mental functions including language, cognition, and emotion [3]. Activity limitations are correspondingly broad, spanning mobility and transfers, self-care such as washing, toileting, and dressing, domestic life, and participation in interpersonal, community, and leisure activities [3]. For these individuals, assistive devices (also referred to as assistive technology) play a critical role in enabling everyday activities to be performed independently, in supporting activities of daily living and social participation, in preventing secondary injuries, and in reducing the physical and psychological burden on caregivers [3,4].

Many people experience substantial functional recovery after stroke, particularly during the early post-onset period, with the majority of gains in activities of daily living consolidated within approximately the first three months [5]. Recovery trajectories nonetheless remain heterogeneous: in a Korean cohort of 4443 people with first-ever stroke, the early recovery curve, the time to plateau, and the pattern of later decline all differed by functional domain, age, initial stroke severity, and stroke type [6]. Over a longer horizon, activity limitations remain relatively stable for several years after onset and then rise again, so that a subset of survivors experience renewed functional decline in the chronic phase [7]. This pattern contrasts with the relatively stable function seen in spinal cord injury after the acute phase, the progressive decline of muscular dystrophy, and the growth-related changes seen in cerebral palsy. Consequently, the devices needed immediately after onset can differ markedly from those needed in the chronic phase, and many devices are needed only for a limited period. This temporal specificity may have implications for how assistive devices are provided, whereas provision systems in South Korea are structured predominantly around ownership-based public funding with fixed durability periods.

In South Korea, assistive devices are provided as public benefits through eight programs administered by four government ministries; over the three years from 2019 to 2021, approximately 15.75 million device provision cases were recorded, amounting to approximately KRW 1.13 trillion [8]. However, according to satisfaction surveys of the assistive device provision program, approximately 41.4% of people who did not use a provided device reported that it was “not suitable for them,” and 14.1% cited incompatibility with their living environment as the primary reason for nonuse [9]. Internationally, roughly 30% of assistive devices have been reported as abandoned [10], and a survey of 749 users of the Italian national health service found that 17.9% had abandoned their assigned device within seven months of receipt, with 40% of that group reporting that they had never used the device [11]. Abandonment and nonpurchase of assistive devices impede rehabilitation and social participation and result in the waste of public resources [10,12,13].

Previous studies on assistive device nonuse have focused primarily on classifying device-related, user-related, and environmental factors across broad disability groups [10,11,13], and large-scale national surveys report aggregate reasons without identifying which specific devices are unsuitable or why [2]. For stroke specifically, information on which devices are used, abandoned, and needed at each stage of the recovery trajectory is critically important for timely provision but remains scarce.

### 1.2. Operational Definitions and Conceptual Framework

The terminology, interview questionnaires, and interview guides used in this study are based on instruments developed and validated in a previous study [14], with modifications to reflect the characteristics of stroke. This research series has applied the same methodology to people with spinal cord injury, muscular dystrophy, and cerebral palsy, and the present study extends the approach to people with stroke. Key terms are operationally defined in this study as follows, drawing on the series’ questionnaire development [14] and standardized assistive-product indicators [15]. Assistive device utilization: regular use of the device at least once within the past three months for a purposeful activity. Assistive device nonuse: any of the following. (i) Possession of a device that has not been used for more than three months, or disposal of the device, without purchasing a replacement device serving the same function. (ii) A device previously used but not used at all at the time of the study. (iii) A device needed for a purposeful activity but not used because it does not fit the user’s body or functional level. Required assistive device (nonpurchase): an assistive device perceived as necessary but not purchased at the time of the study. Change in assistive device utilization: replacement of an assistive device used for the same purposeful activity, including replacement due to wear or breakage, version changes within the same device type, and replacement for usability or safety. Throughout this paper, nonuse is used as the neutral umbrella term covering all three criteria above; abandonment is reserved for nonuse arising from a mismatch between the device and the user or the environment, and discontinuation for nonuse arising from the device no longer being needed.

Interview questionnaires were structured according to the Human–Activity–Assistive Technology (HAAT) model [16], centered on four components: Human, Activity, Assistive Technology, and Context. Post-onset stages for stroke were defined operationally by the research team for this study [17]. The starting point was the staging used in a national assistive device guide for stroke, which divides device use into an acute phase (diagnosis, onset, surgery, and rehabilitation treatment), a recovery phase (return home), and a maintenance phase (community and workplace) [18]. This staging was reviewed with two stroke rehabilitation specialists at the expert advisory meetings held for the research program (Section 2.3). The experts noted that function is typically poorest on admission and improves with rehabilitation, a course consistent with cohort data showing that most gains in activities of daily living are consolidated within the first months after onset [5], and advised collapsing the guide’s phases into two stages that participants could locate reliably in their own histories: the hospitalization stage, defined as “a period of inpatient stay of five or more days per week receiving intensive rehabilitation treatment,” and the home and community reintegration stage, defined as “a period in which the primary residence is the home for five or more days per week, and the individual is preparing for or already engaging in regular social activity following family care or preparation for independent living.” Neither stage is bounded by a fixed time since onset. The stages are defined instead by the setting in which the person spent five or more days per week. A setting-based definition was preferred for two reasons: the length of inpatient rehabilitation varies widely between individuals, so a fixed time boundary would have placed the same clinical situation in different stages for different participants; and because the study relied on recall one to two decades after onset, the move from hospital to home offered a more salient and more reliably recalled anchor than elapsed time [17]. Each device was assigned to the stage in which the participant recalled first using it. One of the experts also advised that, for a clear investigation of device use, participants should be selected by functional level, recommending a Korean version of the Modified Barthel Index (K-MBI) range of 33–80. This band corresponds to the range in which assistive device use is both most prevalent and most differentiated by device type. Its lower bound sits immediately above the threshold for the most severe grade of brain lesion disability under the Korean disability registration system, in which severity for this disability type is determined by the Modified Barthel Index and the most severe grade requires a score of 32 or below together with total assistance for all ordinary activities including ambulation [19]; its upper bound excludes those whose recovery has already rendered devices largely unnecessary. Prospective evidence from people with first-ever stroke supports this framing: those who used no mobility device were less physically disabled than device users, device use was associated with poorer physical functioning but preserved cognition, and device type tracked mobility capacity, with cane users less impaired than walker users and wheelchair use predicted by functional independence [20].

### 1.3. Assistive Device Research in the Korean Stroke Population

According to the 2020 National Survey on Persons with Disabilities [2], people with physical disabilities, brain lesion disabilities, and visual and hearing impairments account for approximately 85% of the roughly 2.44 million device-owning people with disabilities in South Korea, and approximately 80% of devices are purchased through government public funding programs. As shown in Table 1, the most common reason for not using an owned device among people with brain lesion disabilities was that the device was “not suitable for them” (39.9%), followed by “difficult to use due to the environment” (23.1%) and “physical changes after acquiring the device” (22.5%). However, these aggregate findings do not identify which specific devices are unsuitable or why, and thus have not readily translated into practical improvements.

For the stroke population specifically, respondents to the 2020 National Survey who reported a diagnosis of stroke numbered 662, corresponding to a post-stratification weighted national estimate of 183,728 people [2]. Among the reasons for dissatisfaction with devices in use, “poor durability” was the most common, at 52.2% of the 20,066 weighted respondents who answered that item out of the weighted stroke population of 183,728; among the reasons for not purchasing needed devices, “purchase cost” was the most common, at 34.7% of the 41,176 weighted respondents who answered that item (see Section 3.6). These figures indicate that both product quality and financial burden operate as structural barriers for the stroke population, but the survey format cannot reveal the device-specific mechanisms behind them.

### 1.4. International Evidence on Assistive Device Nonuse Relevant to Stroke

The International Classification of Functioning, Disability and Health (ICF) Core Sets for stroke document the breadth of activity and participation domains affected after stroke, including walking (d450), speaking (d330), and self-care activities such as washing (d510), toileting (d530), dressing (d540), and eating (d550) [3], underscoring that assistive device needs after stroke span mobility, self-care, and domestic life. The importance of family support as an environmental determinant of stroke outcomes is also well documented [3,21].

Regarding nonuse, a qualitative interview study of people after stroke and spinal cord injury in Germany explored the provision and use of assistive products and identified factors associated with users’ experiences, including care pathways, care coordination, inter-professional collaboration, professionals’ knowledge and patient information, cost coverage, and approval procedures; deficits were most pronounced among people with more severe mobility impairment [22]. A systematic review of assistive technology acceptance among users with motor disabilities due to spinal cord or acquired brain injuries reported that acceptance is assessed principally through the constructs of satisfaction, ease of use, safety, and comfort [23]. A prospective study of the post-discharge use of assistive devices (grab rails and bathing, toileting, and dressing aids) recommended by occupational therapists during hospitalization found that use after discharge was predicted by users’ perceptions of the characteristics of the device, the presence or absence of anxiety, and their ability to recall device training; intended use after discharge, perceived benefit of the device, negative perceptions about disability, and having had a choice during selection were also strongly related to use, although they did not enter the final model [24]. Classic studies of abandonment similarly identified lack of user involvement in selection, poor device performance, and changes in user needs as principal predictors [10,25].

At the population level, national surveys in Canada and Sweden have documented persistent unmet need for assistive products. In the Canadian Survey on Disability the most common unmet need was for hearing aids, followed by bathroom aids [26], while among older Swedish adults the most frequently used products were for furnishing and home adaptation and the highest perceived unmet need concerned communication aids [27]. The World Health Organization (WHO) has framed the factors preventing needed devices from being acquired and used through the people-centered assistive technology (5P) model, whose five components are People, Policy, Products, Personnel, and Provision [28]. The position paper from the Global Research, Innovation, and Education on Assistive Technology (GREAT) summit argues for a systems-thinking approach in which these components must be addressed together for assistive technology to reach and remain with the user [29]. A scoping review of methods for estimating assistive product supply and demand has called for standardized population-level indicators to make such estimates comparable across settings [15]. Taken together, these frameworks indicate that nonuse and nonpurchase are not matters of individual choice but products of interacting user, product, and system factors. Stroke-specific evidence organized at the device level along the recovery trajectory nonetheless remains lacking, and that is the gap this study addresses.

The two frameworks used in this study operate at different levels and were applied at different points. The HAAT model describes assistive technology use as the fit among a person, an activity, a device, and the context in which the activity is performed [16]. Because the research questions concern which devices were used, set aside, or not obtained, and why, the HAAT model structured RQ1 and RQ2, the interview guide, the device checklist, and the device-by-device record, and it fixed the unit of analysis as an individual device used for a purposeful activity in a particular setting. The HAAT model, however, treats policy, funding, and service delivery only as part of a broad context component, whereas the reasons participants gave for acquiring, replacing, or not purchasing a device frequently concerned exactly these conditions. The WHO 5P framework [28] was therefore adopted for RQ3, because it separates the system-level conditions of access into policy, products, personnel, and provision alongside the user, and because it is the framework through which national and international bodies discuss the provision systems to which the findings of RQ3 are addressed [28,29]. The two frameworks are complementary rather than competing: the HAAT Human and Assistive Technology components correspond broadly to the 5P People and Products components, and the 5P Policy, Personnel, and Provision components disaggregate what the HAAT model groups as Context. The 5P framework did not shape the interview questions or the initial coding. Facilitators and barriers were elicited with open questions for each device within the HAAT Assistive Technology domain (Section 2.3) and coded inductively, and the 5P components were applied only afterwards, as a scheme for organizing the resulting categories (Section 2.4).

### 1.5. Aim and Research Questions

Accordingly, this study conducted in-depth interviews with people with stroke in order to document, at the level of the individual device, the reasons they gave for nonuse and the barriers they reported to purchasing devices they considered necessary. Specifically, this study addressed the following research questions: (RQ1) What types of assistive devices do people with stroke report having used across the two post-onset stages (hospitalization and home and community reintegration), and how do these reported patterns change with functional recovery? (RQ2) What are the device-specific reasons for nonuse and the barriers to nonpurchase? (RQ3) What facilitators and barriers do participants describe as shaping device utilization? The study is exploratory and hypothesis generating: it describes patterns reported retrospectively by a small purposive sample, and does not set out to establish a normative or prescriptive device pathway.

## 2. Materials and Methods

### 2.1. Study Design and Reporting

This study used a qualitative descriptive design in which each participant with stroke was interviewed twice, and the reported device histories were situated against aggregate figures from a secondary cross-sectional analysis of the stroke subsample of a national survey. The primary data are retrospective device histories: for each device, participants recalled whether and when it had been used, discontinued, or replaced, and the reasons for those events. The design is descriptive and hypothesis generating rather than explanatory, and the survey figures serve as national context rather than as a test of the interview findings. Device utilization was documented separately for the two post-onset stages defined in Section 1.2, and reasons for nonuse and nonpurchase were analyzed at the level of the individual device rather than in aggregate.

### 2.2. Participants

As presented in Table 2, the study enrolled 10 community-dwelling adults with stroke, recruited by purposive sampling from the outpatient clinic of the National Rehabilitation Center and through staff referral, in the metropolitan (capital) region of South Korea (IRB No: NRC-2023-01-009). Participants were aged 18 years or older, had prior experience with assistive devices obtained through public funding, personal purchase, modification, or custom fabrication, and had been discharged and were residing in the community at the time of the study. To ensure enrollment of people with substantial device dependency while retaining interview feasibility, a target range of 33–80 on the K-MBI [30] was set on the advice of a stroke rehabilitation specialist at the expert advisory meetings, who identified this band as corresponding to grades 2 to 4 for brain lesion disability under the Korean disability registration system then in force [19], a system in which severity for brain lesion disability is determined by the Modified Barthel Index. This band is also the range in which mobility device use is most prevalent and most differentiated by device type, since people with milder impairment more often use no device at all and device type tracks mobility capacity [20]. The range was applied as a purposive sampling target rather than as a strict eligibility cutoff, and participants were sampled to capture a range of functional levels within it. The K-MBI scores observed at the time of the study ranged from 51 to 82; the one participant who scored 82 was retained because this score remains within the moderate-dependency category of the Modified Barthel Index (61–90) [31]. In terms of global disability, the observed range corresponds approximately to modified Rankin Scale grades 3 to 5 [32]. Sample size adequacy was appraised using the concept of information power [33], under which fewer participants are required when the study aim is narrow, the sample is specific, an established theoretical framework is applied, and the dialogue is rich. All four conditions were met in the present study. The aim was restricted to assistive device histories, the sample was confined to community-dwelling adults with chronic stroke within a defined functional range, the HAAT model structured the instrument, and each participant was interviewed twice for 1 to 1.5 h. The study therefore prioritized depth of inquiry over breadth of sampling. The target of ten participants was fixed at the planning stage of the research program. It was informed by information power and by evidence from customer-needs research that one-to-one interviews with a relatively small number of users identify a large share of the needs that a larger sample would reveal [34], and each participant contributed two interviews rather than one. The sample actually obtained should nonetheless be read alongside this rationale. Although the K-MBI target band was 33–80, eight of the ten participants scored between 74 and 82 and only two scored below 70 (P7, 51; P10, 60), whereas ambulation was more varied (FAC 1–5; Table 3). With ten participants clustered in the upper part of the band, functional level and device type could not be separated analytically, and no comparison of device patterns between functional strata was attempted. The sample was intended to capture a range of device histories within one functional band, not to represent the strata within it, and saturation was not formally assessed. Caregivers, who interact closely with users and are key informants on device use, participated jointly in the interviews where applicable, and information on devices used by caregivers was also collected. All individuals who were approached agreed to participate, and no participant withdrew during the study.

The sample comprised seven men and three women, with a mean age of 65.7 (±5.0) years. The mean age at onset was 51.8 (±5.1) years, with all participants experiencing onset in middle adulthood, and the mean total duration of disability was 13.9 (±4.6) years.

In addition to the K-MBI, functional assessments were conducted with all participants: the Korean version of the Mini-Mental State Examination (K-MMSE), Functional Ambulation Category (FAC), Berg Balance Scale (BBS), Manual Function Test (MFT), and Modified Ashworth Scale (MAS). MFT scores are reported as converted scores, that is, the raw score out of 32 expressed as a percentage. As presented in Table 3, cognition was within or near the normal range for most participants (K-MMSE 27.4 ± 2.5; one participant scored 22), dependency in activities of daily living was moderate (K-MBI 73.7 ± 10.1), and ambulation, balance, upper-limb function, and spasticity profiles were heterogeneous, which is consistent with the functional heterogeneity characteristic of chronic stroke.

These assessments were used only to describe the sample; none served as an outcome measure. The Korean versions of the Modified Barthel Index [30], the Mini-Mental State Examination [35], and the Berg Balance Scale [36] have published evidence of reliability or validity in Korean samples. The FAC [37], the MFT [38], and the MAS [39] were administered in their standard forms, for which reliability and validity have been reported in people with stroke; we did not identify a separately published Korean validation of these three instruments, and their scores should be read with that limitation in mind. Years of formal education were not collected, because the Human domain of the instrument shared across this series [14] does not include education. K-MMSE scores are therefore reported as raw totals without education adjustment. Because K-MMSE performance in Korean older adults varies with age, sex, and education [40], the raw scores cannot be classified against normative cut-offs, and the single score of 22 (P2) cannot be interpreted as indicating cognitive impairment without education data. The K-MMSE was not an eligibility criterion; it was administered to describe cognitive status, and all participants, including P2, completed both interviews.

### 2.3. Data Collection

Each participant was interviewed twice, one week apart, for 1 to 1.5 h per session; all interviews were conducted in Korean at the participant’s home. The two interviewers (E.R., S.-Y.L.) were female researchers at the National Rehabilitation Center, each holding a master’s degree, one in service-convergence design and one in human factors engineering. Both had completed interviewer training and had no relationship with the participants prior to the study. Their disciplinary backgrounds oriented the inquiry toward product usability and the fit between the device and the user’s environment, and conducting the interviews in participants’ homes allowed the settings in which devices were actually used to be observed directly. Prior to the interviews, the purpose, methods, and content of the study were fully explained, and written informed consent was obtained.

The interview questionnaire and a stroke-specific assistive device checklist were developed on the basis of a literature review and refined and validated through the expert advisory meetings described below. The interview guide was organized into four domains aligned with the HAAT model [16]: (1) Human, covering sex, birth year, diagnosis, disability type and severity, time of onset, and comorbid disability; (2) Activity, covering engagement in social activities such as study or work; (3) Assistive Technology, covering device name, duration of use, purposeful activity (ICF d-codes), usage environment (ICF e-codes, such as bathroom, living room, bedroom, office, and public transportation), acquisition route, funding source, product form (off-the-shelf, modified, or custom-made), and facilitators and barriers to use; and (4) Context, covering daily routines and personal assistance services. For the assistive technology domain, questioning was combined with shadowing of device use in the participant’s own home, so that the settings in which devices were actually used could be observed alongside the reported device history.

Expert input was obtained at five advisory meetings held for the brain lesion disability program before data collection, with four specialists in cerebral palsy rehabilitation and two in stroke rehabilitation; together these meetings produced the interview instruments and a separate assistive device checklist optimized for each disability type. The two advisers for stroke, both physiatrists (rehabilitation medicine specialists), one specializing in stroke rehabilitation and one in both stroke and cerebral palsy rehabilitation, were each consulted at a separate meeting. These meetings addressed how the post-onset course of stroke should be divided for a retrospective device history, which functional range should be sampled, and which device categories the stroke checklist should cover, and the advisers’ recommendations were recorded and reviewed by the research team. Both advisers independently recommended dividing the post-onset course into hospitalization and home and community return, the second noting that function is poorest on admission and improves with rehabilitation; the first recommended the K-MBI band of 33–80, and the second advised on the device categories to be covered by the checklist (upper-limb, lower-limb, mobility, and positioning devices), which were added to it. Because the recommendations did not conflict, no formal procedure for resolving disagreement between advisers was required; decisions on adopting each recommendation, and on the operational definitions of use, nonuse, need, and change, were made by consensus within the research team, drawing on prior studies [14,15].

In the first interview, the stroke-specific device checklist was used to document the full history of assistive device utilization, nonuse, and replacement across the two post-onset stages; devices not on the checklist were confirmed and added during the in-depth session. The second interview explored in depth the reasons for use, nonuse, and replacement of each device identified in the first session, together with required but unpurchased devices and the reasons for nonpurchase. By design, the interviews were not audio-recorded; both interviewers instead documented participant responses and observations in detailed field notes during and immediately after each session, and the completed checklist for each participant served as the structured spine of the record. Verbatim transcription is often treated as a prerequisite for interview research, but no reporting standard requires it, and its necessity has been questioned where the analytic aim does not depend on how participants phrase their accounts [41]. A comparative study that produced both audio transcripts and note-based scripts from the same interviews found the two comparable in the detail captured, differing mainly in that notes group topics thematically whereas transcripts preserve conversational order, and concluded that in some circumstances working from notes is the preferable approach rather than a fallback [42]. The conditions that study identifies as necessary, namely experienced and trained interviewers and notes written during and immediately after the session, were met here. The choice also suited the form of the data: the sessions were conducted in participants’ homes and combined completion of the structured device checklist with shadowing of device use across rooms, so the record consists principally of device-by-device entries and observations of the physical setting rather than of continuous narrative. Because no recordings were made, no verbatim quotations are reported; individual cases are identified by participant number (P1–P10), and the consequences of this decision for interpretation are set out in Section 4.8.

The interview schedule was designed to limit the burden of each session. Content was divided between the two visits, so that the first session was confined largely to completing the device checklist and the second to the reasons for use, nonuse, replacement, and nonpurchase of the devices already identified, and sessions were held in the participant’s home, where devices could be shown rather than described. No rest breaks were needed, and no session was shortened, split, or stopped. No participant had severe aphasia, and all were able to respond verbally; where a participant had difficulty with word finding or recall, the stroke-specific device checklist, developed at the expert advisory meetings described above, allowed devices to be identified item by item rather than through free recall or verbal description. Joint interviewing can help reconstruct practices shared within a household, but one party’s account can also shape or displace the other’s [43]. Because participants and caregivers were interviewed together, at the same time and in the same place, any difference between their answers was reconciled during the session, and the reconciled answer was recorded as a single joint response.

### 2.4. Data Analysis

The field notes and the completed device checklists were analyzed by inductive qualitative content analysis within a qualitative descriptive framework [44,45], an approach suited to summarized and structured qualitative records because it stays close to the manifest content of the data rather than seeking latent meaning. The two researchers independently coded the field notes using Atlas.ti 9.1.7.0 software and grouped the coded units into categories, among them the categories of nonuse reasons and of facilitators and barriers, while device counts were tabulated in parallel using a spreadsheet. Because the records are summaries rather than verbatim transcripts, coding was confined to the reasons participants themselves stated for each device, and no inference was drawn from wording, emphasis, or the conduct of the exchange. Coding discrepancies identified after the first interview were discussed between the two coders and, where interpretation remained uncertain, resolved by directly querying the participant at the second interview, providing a form of respondent validation.

To make the path from records to categories traceable, Appendix A (Table A1) gives anonymized examples of summarized field-note entries, the initial codes assigned to them, and the categories under which they are reported. Each reason recorded against a device was also assigned a reason code (for example, functional improvement, use confined to the hospitalization stage, cost, bulk or lack of space, replacement by another device, or poor fit), allowing the reasons underlying each nonuse count to be traced directly. These reason codes were also applied to the devices on which the principle interpretations rest. The records were compiled from sessions in which caregivers could supplement or confirm participants’ answers, and the field notes, like the device-level summary record the reason given for each device without distinguishing the respondent; because any discrepancy between participant and caregiver was reconciled during the session (Section 2.3), each recorded reason is a joint response. Throughout the Results, “participants reported” therefore refers to the joint account of the participant and, where present, the caregiver (Section 4.7).

The analysis was organized into five areas: (1) types of assistive devices used, classified by the Korean assistive device classification system [46] and ICF activity domains, with use tallied by post-onset stage and acquisition type (purchase or rental); (2) assistive devices not used and reasons for nonuse; (3) required assistive devices not purchased and reasons for nonpurchase; (4) assistive device replacement; and (5) facilitators and barriers to device use classified by the WHO 5P framework [28]. Reporting of this study follows the Consolidated Criteria for Reporting Qualitative Research (COREQ) [47]; the completed 32-item checklist is provided in Appendix B (Table A2).

### 2.5. Secondary Cross-Sectional Analysis of the 2020 National Survey

Because the National Survey on Persons with Disabilities does not permit direct comparison with the two-stage classification used in the interviews, a secondary cross-sectional cross-tabulation analysis of the survey raw data [2] was conducted using the duration of disability, computed from the age at which the impairment was first recognized and the current age. Respondents reporting a diagnosis of stroke numbered 662; applying the survey post-stratification weights yielded a national estimate of 183,728 people, and all analyses were conducted on the weighted estimates. Weighted frequency distributions and cross-tabulations were produced with IBM SPSS Statistics for Windows, version 28 (IBM Corp., Armonk, NY, USA), and only descriptive weighted percentages are reported. Four variables were analyzed by duration of disability: needed and used assistive devices, reasons for dissatisfaction with devices, reasons for not purchasing needed devices, and reasons for not using owned devices. Because single-year duration cells contain few unweighted respondents, the duration cross-tabulation was not sufficiently stable to report, and the survey results presented here are aggregate figures for the stroke subsample (Section 3.6). The survey and the interviews draw on different populations and different instruments, so the survey is used here to situate the interview findings within national figures and not as an independent test of them.

## 3. Results

All results below are drawn from joint participant–caregiver accounts, as described in Section 2.4. No participant reported a recurrent stroke, a new musculoskeletal condition, or another new illness during the recall period that had altered their device needs.

Table 4 presents the number of assistive devices used, replaced, and required among the 10 participants with stroke. On average, each participant had experience using 11.2 ± 3.3 types of assistive devices (median 10.5, range 8–20), of which 6.9 ± 3.5 were first used during hospitalization and 4.1 ± 2.0 were first introduced after home and community return (each device type is counted once, at the stage of first use). Participants replaced devices 9.8 ± 7.2 times during the period of use (median 7, range 2–27). The distribution of replacements was right-skewed by one participant (P3) who reported 27 replacements over a 22-year disability duration, so the median provides a more representative summary. The mean number of required but unpurchased assistive devices was 1.3 ± 1.3 (median 1.5, range 0–4).

### 3.1. Assistive Devices Used by Post-Onset Stage

Devices reported at each stage were organized by ICF activity domain and device category, and the resulting distribution is presented in Table 5. Devices commonly used across participants included arm, trunk, and leg exercise devices; canes; manual wheelchairs; standing frames and standing supports; and powered wheelchairs.

During hospitalization, participants first used a mean of 6.9 device types, centered on rehabilitation and in-hospital mobility: standing frames, manual wheelchairs, walkers, exercise devices, pressure-relief devices, and, in cases of severe initial impairment, mobile lifts and portable commodes. Many of these were used as facility or rental devices rather than purchased. After home and community return, participants introduced a mean of 4.1 new device types, oriented toward independent mobility and daily living: canes appropriate to the recovered gait, powered wheelchairs and scooters, vehicle adaptations for driving, bathing aids, non-slip pads, and fixed grab rails and handgrips.

Devices were also switched within the same category as recovery advanced. The cane showed the clearest trajectory: participants progressed from a large quad cane to a small quad or tripod cane during hospitalization, and then to a single-point or lightweight (hiking-type) cane after community return. Similarly, some participants transitioned from an ankle-foot orthosis to a foot orthosis as function improved. The records also note a tendency to rely on the unaffected side rather than on devices where possible; for example, the one participant who had used eating aids during hospitalization reported having learned to eat one-handed with the unaffected hand and no longer needing the device after discharge. That device was subsequently lost and was not replaced.

### 3.2. Assistive Device Nonuse and the Reasons Recorded

Table 6 presents the devices participants had used, the number no longer using each device at the time of the study, and the reasons recorded for stopping use or for replacing a device. Each reason is followed by a reason code, and Table 7 summarizes the reasons recorded for the devices on which the principal interpretations rest. Two clusters of nonuse were distinguished.

The first and larger cluster was discontinuation of devices whose use had been confined to the hospitalization stage. Standing frames and standing supports were used by 9 of the 10 participants, in every case as facility devices during inpatient rehabilitation, and none continued use after discharge (9/9). The reasons recorded for this device were that use had been confined to rehabilitation treatment, that function gradually improved so that the device was no longer needed, that the device was too expensive and too bulky to consider for the home, and, for one participant, that it was still needed after discharge but could not be obtained. Because these reasons were recorded together, the records do not show how many of the nine discontinuations would have occurred on grounds of recovery alone, and discontinuation of this device reflects the end of facility-based access as much as recovery. Manual wheelchairs were used by all 10 participants, largely for rapid movement within the hospital, and 8 of 10 no longer used them; the reasons recorded were functional improvement, deliberate nonuse in order to practice walking, replacement by a powered wheelchair, disposal after the tires deteriorated, and storage without use, and they cannot be assigned one-to-one to the eight participants. Walkers (5/5) were the one device in this cluster for which a single reason was recorded: they had been used for gait training in the early low-function period and no longer matched participants’ functional level once ambulation improved, which meets criterion (iii) of the nonuse definition given in Section 1.2. Arm, trunk, and leg exercise devices (5 of 10 discontinued) were used only as hospital, public health center, or welfare center equipment, none was purchased for home use, and functional improvement, size, and price were all recorded. Portable commodes, mobile lifts, electric beds, and height-adjustable desks followed the same pattern of use confined to the hospitalization stage, with several devices donated to the hospital at discharge. The feature common to every device in this cluster is therefore that its use was confined to one stage; functional improvement was recorded for most of these devices but, with the exception of walkers, alongside cost, space, replacement, or continuing need (Table 7).

The second cluster was nonuse driven by physical incompatibility of body-fitted devices with the ongoing changes in the hemiplegic limb. Ankle-foot orthoses were used by 6 participants and were no longer used by 4 of them; four distinct reasons were recorded for the four nonusers (Table 7). The reasons were mixed: continuous changes in foot shape and function made long-term use difficult and donning and doffing were inconvenient, which meets the definition of abandonment used here, while other participants discontinued the orthosis after function improved or after switching to a cane. Custom corrective shoes were abandoned by both users (2/2) because the shoes lost their shape quickly and had to be replaced repeatedly. Shoulder dislocation-prevention orthoses were abandoned by all three users because the weight of the arm caused neck and shoulder pain. Participants noted that both the number of companies able to custom-fabricate such devices and the funding available for repeated refabrication were insufficient, and that durability-period-based funding is inequitable given individual differences in the pace of physical change.

Additional reasons included environmental incompatibility (bath chairs made narrow bathrooms even narrower, 3/5 discontinued; a mobile lift occupied too much space), product durability and quality problems (pressure-relief cushions leaking air after two years; cane rubber tips wearing out or becoming slippery in rain; grab rails loosening from the wall over time), administrative burden (participants who wished to continue using pressure-relief cushions and grab rails discontinued them because reapplying for support or repurchasing was perceived as cumbersome; one participant lost eligibility after a long-term care grade change), and device–environment mismatch in public infrastructure (powered scooters abandoned because frequent curbs reduced rather than improved mobility; a powered wheelchair replaced after its front wheels repeatedly became caught in subway platform gaps).

### 3.3. Required Assistive Devices Not Purchased and Reasons for Nonpurchase

An average of 1.3 (±1.3) assistive devices per participant were identified as required but not purchased. Figures from the other groups in this research series are shown for descriptive context in Section 3.5 but are not directly comparable. Table 8 summarizes the reasons for nonpurchase by number of cases and the devices involved.

The barrier reported most often was that participants could not find the product they wanted on the market (6 cases), concentrated in exercise, strength, and balance training equipment (3 cases), walking-aid accessories (2 cases), and clothing and footwear (1 case). Participants wanted compact home devices that massage the hand, rotate the ankle, or rotate the arm through 360°, rather than the large facility-type upper and lower limb machines available. The next most frequent barriers were high cost combined with insufficient subsidies (4 cases) and incompatibility with the user’s environment, chiefly the space and size constraints of the home (4 cases). Both again centered on exercise equipment and on clothing and footwear. Difficulty finding information on suitable devices accounted for 3 cases (pressure-relief, fixation, and cooking aids). Two cases involved a psychological reason; in both, participants reported that using the device made them feel bad. Both concerned arm orthoses. The records do not specify whether the feeling concerned the device’s visibility, its discomfort, or what it signified to the participant, so these two cases are reported as psychological reluctance without further interpretation.

Multiple reasons could be reported for a single required device. Reasons are summarized from the interviewers’ field notes and are not verbatim transcriptions; the category of product unavailability reflects participants’ reported inability to find a suitable product and was not verified against a product-market audit.

### 3.4. Facilitators and Barriers to Assistive Device Utilization

Based on the device use histories of the 10 participants, facilitators and barriers to assistive device utilization were derived and classified by the WHO people-centered assistive technology (5P) framework [28], as shown in Table 9. Facilitators included high psychological acceptance and information literacy, financial capacity, active social participation, and the caregiver burden-relieving effect of devices; product-side facilitators included good compensation of bodily function, robustness, safety, aesthetics, environmental fit, and convenient operation. Barriers mirrored these dimensions: low psychological acceptance and information access; desired products missing from funded item lists or not commercialized; high copayments for high-function devices and insufficient support for custom-made devices; devices mismatched to a continuously changing body or to the home environment; frequent breakdowns with difficult after-sales service; recommendations by professionals that did not consider the user’s individual environment; rental period limits; and a shortage of channels for obtaining device information.

### 3.5. Comparison Across Disability Types in the Research Series

Table 10 places the present figures alongside those obtained for spinal cord injury, muscular dystrophy, and cerebral palsy with the same interview methodology in this research series [17,48]. The comparison is descriptive only. The groups differ in size, in age, and in duration of disability, and because device types used, replacements, and required devices are lifetime counts that accumulate with time since onset, the figures are not directly comparable between groups, and no ranking or group difference is inferred from them. With this caveat, the number of device types used was of similar magnitude across groups (means of approximately 10–12), whereas replacement counts and counts of required but unpurchased devices varied more widely.

### 3.6. Secondary Cross-Sectional Analysis of the 2020 National Survey (Stroke Subsample)

Cross-tabulation by duration of disability did not yield stable estimates. Single-year duration cells contained too few unweighted respondents to support inference, and because a cross-sectional survey of prevalent cases contains proportionally more respondents with long disability duration, any apparent clustering by duration could not be separated from sample composition. The survey results are therefore reported for the stroke subsample as a whole. The two attitudinal items use a different base. Of the weighted stroke population of 183,728, the dissatisfaction item was answered by 20,066 weighted respondents, among whom “poor durability” was the most common reason at 52.2%; the nonpurchase item was answered by 41,176 weighted respondents, among whom “purchase cost” was the most common reason at 34.7% [2].

These population-level findings are broadly consistent with the interview results: durability problems (worn cushions, deteriorated tires, loosening grab rails, wearing cane tips) recur as device-level dissatisfaction, and cost operates as a structural nonpurchase barrier at the national scale even though, at the individual level, the barrier articulated most often in this study was the inability to find a suitable product on the market. Because the two sources cover different populations and use different instruments, this agreement is best read as convergence between two independent descriptions rather than as confirmation of the interview findings. The survey format cannot identify which devices are involved or why, which is the gap the device-level interview findings reported above are intended to fill.

## 4. Discussion

This study documented retrospective accounts of assistive device utilization, nonuse, replacement, and nonpurchase across two post-onset stages among 10 community-dwelling adults with stroke, and identified the device-specific reasons participants gave for nonuse and the barriers they reported to nonpurchase. To our knowledge, this is the first study in South Korea to conduct stage-specific, device-level analysis of assistive device nonuse and nonpurchase among people with stroke using a dual-interview qualitative design informed by the HAAT model, set against a secondary cross-sectional analysis of the national survey stroke subsample. The findings are exploratory: they describe what a small purposive sample recalled, and the interpretations offered below are hypotheses for prospective testing rather than established mechanisms. Throughout this section, the accounts discussed are joint accounts of participants and, where present, caregivers (Section 4.7).

### 4.1. Stage-Limited Devices and the Uncertain Contribution of Recovery to Discontinuation

The largest cluster of nonuse in this study concerned devices whose use had been confined to the hospitalization stage. This pattern is reconstructed from participants’ retrospective accounts and should be read as a description of what they reported rather than as an observed trajectory. Standing frames (9/9), walkers (5/5), and manual wheelchairs (8/10) had the highest proportions of nonuse, but these figures count discontinuation, not discontinuation caused by recovery. Functional improvement was the only reason recorded for walkers; for standing frames and manual wheelchairs it was recorded alongside cost, bulk, replacement by another device, and, in one case, continuing unmet need, and the records do not allow these reasons to be separated for individual participants (Table 7). The interpretation that these devices served as temporary supports for a recovering function is therefore well supported for walkers and only partly supported for the other two devices. These proportions are conditional on the functional band sampled here. Every participant retained some ambulatory capacity (FAC 1–5, mean 3.6; Table 3), whereas among people who do not regain independent ambulation the manual wheelchair is a permanent rather than a stage-limited device. Discontinuation associated with recovery therefore describes a trajectory available to those who recover rather than a property of the device itself. Powered scooters were also discontinued by both users, but for a distinct reason. Frequent curbs in the built environment reduced rather than improved mobility, so these devices are treated under environmental mismatch in Section 3.2 rather than as discontinuation associated with recovery. Earlier reports in this series described nonuse in spinal cord injury as driven mainly by physical incompatibility in a stable body [48], in cerebral palsy by growth-driven refitting [17], and in muscular dystrophy by progressive functional decline [48]; in the present sample, some nonuse appears instead to have accompanied successful rehabilitation, although the groups were not compared directly (Section 3.5). The distinction is terminological as well as substantive. Where a device is set aside because the user no longer needs it, discontinuation describes the event more accurately than abandonment, which carries an implication of service or product failure; where cost, space, or continuing need were recorded alongside improvement, as for standing frames, neither term alone describes the event adequately [11]. This pattern has precedent in the wider assistive technology literature. In a post-discharge follow-up of 47 adults with mixed diagnoses discharged from an inpatient rehabilitation unit, only 86 of 128 prescribed devices were still in use at three months, and quad canes, walkers, and manual wheelchairs were among those most frequently abandoned, at 43%, 36%, and 36%, respectively. The most common reason given was that the device was no longer needed, and functional improvement accounted for approximately half of the nonuse observed [49]. The remaining nonuse in that study was attributed not to recovery but to divergence between clinicians’ and users’ judgments of the usefulness and appearance of a device [49], which parallels the psychological reluctance recorded for two arm orthoses (Section 4.4). That cohort also comprised mixed diagnoses, so it cannot establish whether discontinuation associated with recovery is specific to stroke. Stage dependence is visible earlier in the trajectory as well. In a one-year randomized follow-up of people aged 70 years and older after acute stroke, simple and inexpensive devices were prescribed predominantly within the first three months, and prescription patterns differed between the first three months and the remainder of the first year [50]. The present findings are consistent with these observations and add device-level and stage-level detail from a stroke-only sample, although, being retrospective and drawn from ten participants, they cannot establish the timing of discontinuation independently of recall. These observations raise a hypothesis about provision rather than a conclusion. Participants described purchasing, storing, donating, or discarding devices they no longer needed, which suggests that ownership-based provision may fit stage-limited devices poorly; whether rental-based provision would fit them better, and whether it would improve outcomes or reduce waste, cannot be tested with ten retrospective accounts. The hypothesis concerns the timing and modality of provision rather than its overall volume. That all in-hospital uses of standing frames (9/9) and of arm, trunk, and leg exercise devices (10/10) were facility or rental devices shows that such arrangements already exist in the hospital setting, but it does not show that users would prefer or benefit from them after discharge.

### 4.2. Device Switching Within a Category as a Marker of Recovery

Device switching within the same category appeared to track recovery in participants’ accounts. It was clearest in the cane trajectory, which progressed from a large quad cane to a small quad or tripod cane, then to a single-point cane, and finally to a lightweight hiking-type cane, and it was also evident in the transition from ankle-foot orthoses to foot orthoses or canes. This “downgrading” trajectory is the mirror image of the “escalation” trajectory observed in progressive conditions and the “refitting” trajectory in growth, and, if confirmed prospectively, it would suggest that provision for stroke may need to anticipate sequential, time-limited device needs rather than single definitive prescriptions. The prospective evidence that post-discharge use of recommended devices depends on users’ perceptions and intended use of them [24] is consistent with the value of reassessment at the hospital-to-home transition rather than a one-time assessment at prescription [10,25], a proposition this study did not test.

### 4.3. Body-Fitted Devices and the Limits of Durability-Period Funding

A countervailing cluster of nonuse arose from body-fitted devices that could not keep pace with ongoing changes in the hemiplegic limb. Ankle-foot orthoses (no longer used by 4 of 6) and custom corrective shoes (2/2) required repeated refabrication as foot shape, tone, and function changed, yet participants reported that both custom-fabrication capacity and funding for repeated refabrication are insufficient, and that fixed durability-period funding is inequitable given individual differences in the pace of change. This converges with the German qualitative finding that cost coverage and approval procedures are among the factors shaping assistive-product provision after stroke and spinal cord injury [22], and parallels the growth-adaptive funding problem identified for cerebral palsy in this series [17]. Whether a physician-prescription-based pathway that funds refabrication according to documented physical change, rather than elapsed time, would serve such devices better is a hypothesis that merits evaluation; the present data describe the problem participants reported but cannot establish the remedy.

### 4.4. Psychological Reluctance and Compensation with the Unaffected Side

Participants’ accounts also recorded compensation with the unaffected side in place of devices. They learned to eat one-handed instead of continuing to use eating aids, and deliberately abstained from wheelchairs in order to practice walking. Psychological reluctance also appeared explicitly among the reasons for nonpurchase. Two participants declined arm orthoses, reporting that using the device made them feel bad; the records do not specify whether this concerned visibility, discomfort, or what the device signified, and no other instance of stigma-related reasoning was recorded in this sample. These observations align with evidence that acceptance of assistive technology in acquired brain injury depends on perceived benefit relative to psychological and social costs [23] and with the classic finding that devices are abandoned when their relative advantage does not outweigh their burdens [10,25]. They are also consistent with person-centered matching approaches, which hold that sustained device use depends on the fit between the user’s characteristics, preferences, and psychosocial readiness on the one hand and the attributes of the technology on the other, rather than on functional need alone [4]. A scoping review of equipment provision after stroke identified this same tension, between equipment provision and prevailing models of stroke rehabilitation, as one of four recurring themes across the literature, alongside the effect of stroke-specific impairments on how equipment is used and taught [51]. That review located the recurring problems in under-provision and insufficient training rather than in over-supply, a framing that complements rather than contradicts the argument in Section 4.1, which concerns when and how devices are provided rather than how many. One possible implication, resting on few cases, is that device provision and the goal of restoring function may be experienced as competing objectives after stroke; whether counseling that presents devices as supports for recovery, or peer experience sharing among users with the same disability type, would lower such barriers is a question for future work.

### 4.5. The Shape of Unmet Need After Stroke

The number of required but unpurchased devices was low (mean 1.3). The corresponding figures from other groups in the series [48] are not directly comparable (Section 3.5), so the low count is interpreted here within this sample only. Several mechanisms may plausibly combine to produce it. Functional recovery genuinely reduces residual need, and compensation with the unaffected side substitutes for devices. With a mean of 13.9 years since onset and onset in middle adulthood, participants may also have had limited awareness of newer devices. The content of the unmet needs is nonetheless informative. The barrier reported most frequently was that participants could not find a suitable product on the market (6 cases), concentrated in compact home exercise devices for the hand, ankle, and arm, which are the functional domains affected by hemiplegia, alongside walking-aid accessories and adapted clothing and footwear. At the national scale, cost was the leading nonpurchase reason (34.7%) and durability the leading dissatisfaction (52.2%) in the stroke subsample [2]. Taken together, these findings are compatible with unmet need after stroke being constrained on the demand side, through compensation with the unaffected side and limited awareness of newer devices, as well as on the supply side, through products participants could not find; if so, expanding subsidies alone might not close the gap. This possibility is consistent with the WHO global report, which identifies inadequate product range, cost, and awareness as the principal barriers to access worldwide [52].

Device need after stroke is not resolved simply by the passage of time since onset, and three converging lines of evidence indicate why it can persist, and re-emerge, well into the chronic phase. First, functional status does not remain stable once formal rehabilitation ends. Approximately one in five stroke survivors shows a clinically meaningful decline in mobility between one and three years after stroke, with inactivity, cognitive problems, fatigue, and depression at one year as predictors [53]. This does not contradict the pattern of stage-limited discontinuation described in Section 4.1. In that cohort roughly four in five did not decline, and the interviews reported here cover the hospitalization and early home-return stages, so later deterioration falls largely outside the window they describe. Devices prescribed during the recovery period may therefore cease to fit the user’s body and environment years later, which converges with the aggregate national finding in Table 1 that unsuitability of the product at 39.9% and physical change after acquisition at 22.5% together account for over 60% of nonuse among people with brain lesion disability. Second, unmet need does not diminish with time since onset; a systematic review of 32 quantitative studies reported an unmet-need prevalence of 62% at six months and 81% at two years after stroke [54], and a survey of community-dwelling stroke survivors in the Republic of Korea documented substantial unmet needs for rehabilitative management well beyond the acute phase [55]. These prevalence figures index a broader construct, covering information, psychological, secondary-prevention, and rehabilitation service needs, than the device-specific count of required but unpurchased items reported in this study, so the two sets of findings are complementary rather than contradictory. Third, the Korean provision system reimburses each device type only once within a fixed durability period, which ranges from six months to six years by item and is five years for manual wheelchairs and six years for powered wheelchairs [56], so that people a decade or more after onset have entered a second or third replacement cycle. This offers one explanation for the prominence of durability and purchase cost among the reasons reported in the national survey (Section 3.6), and it is consistent with the hypothesis raised in Section 4.3 of provision keyed to documented physical change rather than to elapsed time. These mechanisms are hypotheses generated by cross-sectional data rather than tested pathways, and they require longitudinal confirmation.

### 4.6. Hypotheses for the Assistive Technology Provision System

The facilitator and barrier analysis organized by the 5P framework [28], read alongside the systems-thinking approach articulated at the GREAT summit [29], suggests five hypotheses that could be tested in South Korea’s provision system. They are derived from ten retrospective joint accounts and are offered for evaluation, not as recommendations. First, rental-based provision for stage-limited, high-cost devices with a short use cycle, such as standing frames, exercise equipment, manual wheelchairs, and electric beds, might lower access barriers and reduce waste. Second, prescription-based flexible provision might fit orthoses and other body-fitted devices that require repeated refabrication better than fixed durability-period funding. Third, integrated information channels that consolidate device and funding information currently dispersed across ministries, together with case management across the recovery trajectory, might reduce the information barriers participants described. Fourth, reimbursed device training, and professional training that takes account of the user’s environment, might increase the use of provided devices; trial evidence indicates that additional home-based training in device use after discharge yields higher rates of device use and user satisfaction among older adults with stroke [57]. Fifth, development of the product categories participants could not find, namely compact home exercise devices, walking-aid accessories, and adapted clothing and footwear, together with arrangements for trying a device before acquiring it, might address part of the unmet need recorded here. Each would require evaluation in larger and prospective studies before adoption, consistent with the WHO position that assistive technology enables healthy, productive, independent, and dignified participation in society [52].

### 4.7. Joint Participant–Caregiver Accounts

Caregivers took part in the interviews where applicable and could supplement or confirm participants’ answers. This arrangement was chosen because caregivers are key informants on device acquisition and use and because recall of events one to two decades earlier was expected to benefit from a second informant. It also has consequences for interpretation. The presence of a caregiver may systematically shape a participant’s account: participants may defer to a caregiver’s version of events, understate difficulties or needs that would imply additional caregiving, or overstate their independence, and caregivers may describe devices chiefly from the standpoint of their own caregiving tasks. Joint interviews can help reconstruct practices that a household shares, such as acquiring, storing, and discarding devices, but one party’s account may shape or displace the other’s [43]. Proxy information after stroke is also more reliable for physical and activity-of-daily-living domains than for psychological and communication domains [58], and the reasons for discontinuation and the barriers to purchase reported here include psychological reasons. Because the field notes did not distinguish the respondent and any disagreement between participant and caregiver was reconciled during the session, each recorded answer is a joint response, and the two sources cannot be separated in the present analysis. The findings are accordingly reported, and should be read, as joint participant–caregiver accounts rather than as participants’ own perspectives, and interpretations of participants’ subjective reasons, such as psychological reluctance, are correspondingly tentative. The count of required but unpurchased devices may be affected in either direction, since caregivers may add needs that the participant did not voice or discourage the voicing of needs that would require further purchases.

### 4.8. Limitations

The principal limitation of this study is that the stage-specific pattern of device use is reconstructed retrospectively. Participants had a mean disability duration of 13.9 ± 4.6 years, so the hospitalization stage and the early period after home return lay one to two decades in the past for most of them. Recall bias is therefore likely to bear on precisely the quantities on which the central findings rest, namely when each device was used, when and why it was discontinued or replaced, and which devices were needed but not obtained; a sequence that was gradual or irregular at the time may be smoothed or reordered in memory, and the device market participants encountered may differ from the present one. The device checklist and the two-interview structure were designed to anchor recall to concrete objects rather than to general impressions, and the second interview was used to confirm and correct the history built in the first, but neither procedure substitutes for prospective observation. The stage-specific trajectory described here should accordingly be treated as a hypothesis to be tested by prospective follow-up of people currently in the hospitalization stage or newly returned home, and not as an established course of device use after stroke.

A second consideration concerns the form of the qualitative record. Because the interviews were not audio-recorded, the analysis rests on the interviewers’ field notes and the completed device checklists, so the data support description of the reasons participants stated for each device but not close analysis of how those reasons were expressed. This was a deliberate choice matched to the form of the data (Section 2.3), and note-based records have been shown to capture detail comparable to transcripts where interviewers are trained and notes are written promptly [42]. That same work cautions that poorly documented notes yield poor data, which is why independent coding by two researchers, cross-checking of the field notes, and respondent validation at the second interview were built into the design. Two constraints nonetheless remain: interviewer selection and interpretation at the point of note-taking cannot be excluded, and no verbatim quotations can be offered as evidence for the categories reported. The analysis was accordingly confined to manifest content (Section 2.4). A study in this series aimed at how participants articulate their experience, rather than at which devices they used and why, would require audio recording with verbatim transcription.

The secondary national survey analysis is likewise contextual rather than confirmatory. It cannot reproduce the stage-specific analysis, since the duration cross-tabulation was not stable enough to report (Section 3.6), and its respondents differ from the interview sample in functional composition and in the way the questions were put. The interview sample was confined to a single K-MBI band whereas the survey covers the full severity spectrum of registered stroke disability. The agreement between the two sources on durability and cost should therefore be read as convergence between two independent descriptions, not as validation of the interview findings.

Finally, the device distribution presented in Table 5 is a record of what these ten participants reported using at each stage. It is not a clinical recommendation, and it should not be read as specifying which devices ought to be provided at which stage. Establishing such a pathway would require a formal consensus procedure among multiple clinicians and users together with prospective validation, which was outside the scope of this study.

The sample also constrains transferability. This study enrolled 10 people with stroke within a K-MBI range of 33–80 recruited in a single region. The adequacy of the sample size was appraised using the concept of information power [33]; nevertheless, transferability of the findings to the broader stroke population is limited, and the functional inclusion range excludes both people with the most severe dependency and those with near-complete recovery. The 10 participants also did not fill the target band evenly: observed K-MBI scores ranged from 51 to 82, so the lower half of the intended 33–80 range is not represented, and no functional stratum within the band contains more than a few participants. This range was set on the advice of a stroke rehabilitation specialist consulted for this study. The lower bound was intended to enroll people with substantial device dependency who nonetheless retained sufficient functional and communicative capacity to recount their own device histories across two interviews, and the upper bound to exclude those whose recovery had already rendered assistive devices largely unnecessary. People who were bedridden or totally dependent were accordingly not enrolled. In that group most device use is caregiver mediated, since positioning systems, transfer lifts, pressure-relief mattresses, and bath stretchers are operated by caregivers rather than by the user, so the categories of use, nonuse, and articulated need that organize this study cannot be elicited from the user directly, and the account would have had to rest on proxy report. Proxy reports in stroke are reliable for physical and activity-of-daily-living domains but considerably less so for psychological and communication domains [58], which are precisely the domains through which nonuse and unmet need were interpreted here. The device categories most relevant to people with the most severe dependency are therefore absent from these findings, and a separate, caregiver-centered design is required to address them. Cognition was also within or near the normal range for most participants (K-MMSE 27.4 ± 2.5; one participant scored 22), and no participant had severe aphasia, so the near-absence of communication and cognitive assistive devices in the findings should not be generalized to the full stroke population, in which communication and cognitive impairments are common [3]. Future studies should include wider functional ranges and participants with communication support needs.

Further constraints concern the descriptive measures, the recall period, and the attribution of reasons. Years of education were not recorded, so K-MMSE scores could not be adjusted for education or compared with normative cut-offs, and three of the functional measures (FAC, MFT, and MAS) lack a separately published Korean validation (Section 2.2); these measures describe the sample only and do not bear on the main findings. No intervening health events such as recurrent stroke or new musculoskeletal conditions were reported (Section 3), although this rests on participants’ and caregivers’ recall rather than on medical records. Finally, the summary records do not attribute each reason for discontinuation to a specific participant, so the number of discontinuations explained by recovery alone could not be established, and the interpretation of stage-limited nonuse is limited accordingly (Table 7).

The sex distribution was also male-skewed (7 of 10), and all participants experienced onset in middle adulthood; sex- and age-at-onset-related differences in device utilization could not be examined. As discussed in Section 4.7, caregiver perspectives may have influenced the reporting of unmet needs; as in the cerebral palsy study in this series [17], the nonpurchase count (mean 1.3) should therefore be interpreted as a lower-bound estimate of actual unmet need. One further ambiguity concerns attribution. Participants’ reports that a device was no longer needed cannot be fully separated from insufficient training in its use; the trial evidence that post-discharge training raises device use and satisfaction [57] indicates that some discontinuation interpreted here as recovery-driven may instead reflect unsupported adoption.

## 5. Conclusions

In retrospective joint participant–caregiver accounts from ten community-dwelling adults with chronic stroke, nonuse of assistive devices was concentrated among devices whose use had been confined to the hospitalization stage. Functional improvement was among the reasons recorded, but only for walkers was it the sole reason; for standing frames and manual wheelchairs it could not be separated from cost, bulk, replacement, and continuing need. A second cluster of nonuse involved body-worn devices that did not keep pace with a changing hemiplegic limb, and the barrier to purchase reported most often was inability to find a suitable product.

These findings describe what a small purposive sample recalled; they do not establish a course of device use or support policy conclusions. Whether rental provision for stage-limited devices, prescription-based provision for body-fitted devices, and targeted product development would serve people with stroke better than ownership-based provision with fixed durability periods are hypotheses to be tested in prospective, audio-recorded studies of people currently in the hospitalization and early home-return stages.

## Figures and Tables

**Table 1:** Reasons for not using owned assistive devices among people with physical and brain lesion disabilities in South Korea (%).

Reasons for Not Using Assistive Devices	The Product Is Not Suitable for Them	Difficult to Use Due to Environment	Physical Changes After Acquiring the Device	Unable to Repair Due to Malfunction	Do Not Know How to Operate It	Other	Total
Physical disability	42.8	21.5	14.7	9.5	3.4	8.2	100.0
Brain lesion disability	39.9	23.1	22.5	3.4	2.6	8.5	100.0

Source: 2020 National Survey on Persons with Disabilities [2].

**Table 2:** Study participants (stroke, *n* = 10).

Participant	Sex	Diagnosis	Age (Years)	Age at Onset (Life Stage)	Total Duration of Disability (Years)
P1	M	Brain lesion (stroke)	71	52 (Middle adulthood)	19
P2	M	Cerebral hemorrhage	63	50 (Middle adulthood)	13
P3	M	Cerebral infarction	74	52 (Middle adulthood)	22
P4	F	Brain lesion (stroke)	61	43 (Middle adulthood)	18
P5	F	Cerebral hemorrhage	66	59 (Middle adulthood)	7
P6	M	Cerebral infarction	66	54 (Middle adulthood)	12
P7	M	Brain lesion (stroke)	65	50 (Middle adulthood)	15
P8	M	Cerebral hemorrhage	56	45 (Middle adulthood)	11
P9	F	Cerebral infarction	67	57 (Middle adulthood)	10
P10	M	Cerebral hemorrhage	68	56 (Middle adulthood)	12
Mean (SD)			65.7 (±5.0)	51.8 (±5.1)	13.9 (±4.6)

**Table 3:** Functional assessment of participants (*n* = 10).

Participant	K-MMSE (/30)	K-MBI (/100)	FAC (/5)	BBS (/56)	MFT (Converted Score)	MAS
P1	25	78	5	49	84.375	U/E: G1+, L/E: G1+
P2	22	74	4	23	84.375	U/E: G3, L/E: G1+
P3	27	79	4	50	50	U/E: G1+, L/E: G1
P4	28	74	1	21	25	U/E: G0, L/E: G0–G1
P5	30	80	4	53	15.625	U/E: G0, L/E: G0
P6	30	79	5	49	12.5	U/E: G1, L/E: G1
P7	29	51	2	28	90.625	U/E: G1+, L/E: G1
P8	29	80	4	48	53.125	U/E: G1–G1+, L/E: G1
P9	27	82	4	49	50	U/E: G1, L/E: G1
P10	27	60	3	29	25	U/E: G1+, L/E: G1
Mean (SD)	27.4 (±2.5)	73.7 (±10.1)	3.6 (±1.3)	39.9 (±12.9)	49.1 (±29.5)	

MFT scores are converted scores, calculated as the raw score out of 32 expressed as a percentage; the values therefore fall on multiples of 3.125. K-MMSE, Korean Mini-Mental State Examination; K-MBI, Korean Modified Barthel Index; FAC, Functional Ambulation Category; BBS, Berg Balance Scale; MFT, Manual Function Test; MAS, Modified Ashworth Scale; U/E, upper extremity; L/E, lower extremity.

**Table 4:** Number of assistive devices used, replaced, and required among people with stroke (*n* = 10).

Participant	Device Types First Used During Hospitalization	Device Types First Introduced After Home and Community Return	Total No. of Device Types Used	Total No. of Device Replacements (Cases)	No. of Required Devices
P1	5	4	10	6	0
P2	3	6	9	6	0
P3	15	5	20	27	2
P4	6	5	11	16	0
P5	7	4	11	11	1
P6	5	2	8	5	2
P7	10	0	10	11	2
P8	4	7	11	2	4
P9	6	4	10	6	2
P10	8	4	12	8	0
Mean (SD)	6.9 (±3.5)	4.1 (±2.0)	11.2 (±3.3)	9.8 (±7.2)	1.3 (±1.3)

The two stage columns count each device type once, at the stage in which it was first used: a device first used in hospital and continued at home (for example, a cane obtained before discharge) appears only in the hospitalization column, so the home and community column counts device types newly introduced after return home, not all devices in use at home. The value of 0 for P7 therefore means that no new device type was introduced after return home, not that P7 used no devices at home. Total device types are counts of distinct device types across the whole post-onset period. For two participants the total exceeds the sum of the two stage-specific counts by one device, because that device was recorded in the device history without an assigned post-onset stage; the stage-specific columns therefore do not always sum to the total.

**Table 5:** Assistive devices reported by people with stroke, by post-onset stage.

ICF D	Device Category	Hospitalization	Home and Community Return
D4 Mobility	Seating and positioning aids	Standing frame and standing support (R); body-worn grasping device (hand); foot orthosis, ankle-foot orthosis; custom corrective shoes for deformity/function; seat belts, belts, and harnesses for body stabilization (shoulder, scapula, waist, etc.)	Body-worn grasping device (hand); foot orthosis, ankle-foot orthosis; custom corrective shoes for deformity/function; seat belts, belts, and harnesses for body stabilization; elbow orthosis (N)
	Wheelchairs and mobility aids	Manual wheelchair (R); mobile lift (R); walker; cane (large quad → small quad/tripod)	Cane (small quad/tripod → single-point → hiking-type); powered wheelchair, powered scooter; powered wheelchair accessories (lap board); wheelchair/user protection devices against sunlight or precipitation (umbrella holder) (N); others (phone charger, etc.) (N)
	Vehicle-related aids	—	Vehicle accessories/adaptations for steering control (steering knob, etc.); vehicle accessories/adaptations for speed control (accelerator pedal, etc.)
D5 Self-care	Daily living aids	Eating and drinking aids; portable commode; pressure-relief cushion, pressure-relief mattress; arm, trunk, and leg exercise devices (upper and lower limb equipment) (R); shower/bath chair; bath towels, sponges, and brushes with fixation devices or handles	Pressure-relief cushion; arm, trunk, and leg exercise devices (upper and lower limb equipment); shower/bath chair; bath towels, sponges, and brushes with fixation devices or handles; arm, trunk, and leg exercise devices (hand, ankle, arm-rotation exercise, etc.) (N); buttoning and fastening aids (shoelace aids) (N)
D6 Domestic life	Daily living aids	Bed and detachable bed board/powered adjustable mattress support platform (R); lifting and tilting system (height-adjustable desk) (R)	Food and beverage preparation aids (jar/bottle fixation aids) (N)
	Environmental and housing modification aids	—	Personal environment control software; non-slip pads; fixed grab rails and handgrips

Devices without a marker were mainly purchased for use; (R) indicates devices used mainly through rental or facility loan; (N) indicates devices reported as needed but not acquired. The table records the devices these ten participants reported at each stage and is descriptive; it is not a clinical recommendation or a prescriptive device pathway. ICF, International Classification of Functioning, Disability and Health.

**Table 6:** Use and cessation of use of assistive devices, with the reasons recorded for stopping use or for replacement, with reason codes (*n* = 10).

Device Classification	Assistive Device	No. of Users (Purchase/Rental)	No. Who Stopped Using the Device	Reasons for Stopping Use or for Replacement
1 04 33 Pressure-relief devices	Pressure-relief cushion	5 (5/0)	1	Being a rubber product, it wore out and leaked air after more than two years, making it difficult to use; wanted to continue using it but found reapplying for support burdensome [D, A]
	Pressure-relief mattress	1 (0/1)	1	Used only in the hospital ward during the period of poorest function [H]
1 04 48 Exercise, strength, and balance training equipment	Arm, trunk, and leg exercise device (upper and lower limb equipment)	10 (0/10)	5	Function improved, so no longer used; rented one but returned it because the house was small and it was cumbersome; too large and too expensive to purchase for home use [R, S, C, H]
	Arm, trunk, and leg exercise device (vibration exerciser)	1 (1/0)	0	—
1 09 24 Urinary management devices	Portable commode	1 (1/0)	1	Used during hospitalization when severely impaired; donated at discharge [H]
2 06 06 Arm orthoses	Body-worn grasping device (hand)	2 (2/0)	0	The hand kept deforming, so the device was discarded and repurchased [F] (replacement)
2 06 12 Leg orthoses	Ankle-foot orthosis	6 (6/0)	4	The ankle part was bothersome when walking, so the user cut it, but it became uncomfortable and was discarded; stretched out, so discarded; function improved, so no longer used; no longer used after switching to a cane [F, D, R, P]
	Custom corrective shoes for function	2 (2/0)	2	The shoes kept stretching, requiring repeated replacement; even when purchased, they stretched quickly, so the user stopped buying them [D, F]
	Foot orthosis	5 (5/0)	1	The sole area was too hard, causing foot pain [F]
3 05 36 Training aids for changing and maintaining body position	Standing frame and standing support	9 (0/9)	9	Used only during rehabilitation hospital treatment; too expensive and too bulky to consider purchasing for the home; still felt it was needed after discharge; legs hurt considerably; function gradually improved, so the device was no longer needed [H, C, S, U, F, R]
3 09 07 Body stabilization aids	Seat belts, belts, and harnesses for body stabilization	1 (1/0)	1	Physical function improved, so no longer used [R]
	Shoulder dislocation-prevention orthosis	3 (3/0)	3	The arm was too heavy, causing considerable neck and shoulder pain [F]
3 09 33 Washing, bathing, and showering aids	Shower/bath chair	5 (5/0)	3	The bathroom was small, and the bath chair made it even narrower, so it was discarded [E]
	Bath towels, sponges, and brushes with fixation devices or handles	4 (4/0)	1	Purchased at a vendor in the hospital basement because it looked good, but the shoulder could not be raised, so it was used once and never again [F]
3 12 03 Single-arm operated walking aids	Cane	10 (10/0)	1	Switched to a simpler cane as function improved; hard rubber tips were slippery on rainy days, so no longer used; the rubber wore out and canes sometimes broke; a cane bought by a family member did not fit the user’s function and was discarded; could not purchase after the long-term care grade changed from 3 to 4 and support was no longer available [R, P, D, F, A]
3 12 06 Two-arm operated walking aids	Walker	5 (4/1)	5	Did not fit the user’s functional level, so it could not be used [R]
3 12 12 Vehicle accessories and adaptations	Vehicle accessories/adaptations for steering control	1 (1/0)	0	—
3 12 22 Manual wheelchairs	Standard manual wheelchair	10 (2/8)	8	Kept in storage just in case, but not used; after discharge, deliberately did not ride it in order to practice walking; used during hospitalization to move quickly to the therapy room, but discarded after discharge; rarely used after purchasing a powered wheelchair; discarded when the tires deteriorated; function improved [R, H, P, D]
3 12 23 Powered mobility devices	Powered wheelchair	8 (8/0)	0	The front wheels kept getting caught in subway platform gaps, so the device was discarded and replaced [E] (replacement)
	Powered scooter	2 (2/0)	2	Korean roads have many curbs, which actually reduced mobility [E]
3 15 09 Eating aids	Eating and drinking aids	1 (1/0)	1	Trained one-handed eating with the unaffected hand and no longer needed the device; the device was subsequently lost and not replaced [M, L]
3 18 10 Seating furniture accessories	Chair-mounted lap tray (lap board)	1 (1/0)	1	After losing it, the price was too high to repurchase [L, C]
3 18 12 Beds and bed equipment	Bed and detachable bed board/powered adjustable mattress support platform (electric bed)	3 (1/2)	2	Participants who used the device during hospitalization did so as a rental and stopped once function improved; also not used because the powered function broke down and because the attached safety rail was inconvenient [H, R, D, F]
3 18 15 Furniture height adjustment aids	Lifting and tilting system (height-adjustable desk)	1 (0/1)	1	The hospital bed table was too small, so this was additionally purchased and used, then donated at discharge [H]
3 18 18 Support handles and grab rails	Fixed grab rails and handgrips	5 (5/0)	1	It had become worn, and reinstalling after moving seemed bothersome, so it was left at the previous house; even when screwed into the wall, it loosened over time and had to be discarded or replaced; repurchasing was felt to be troublesome and applying for support burdensome [D, A]
3 18 30 Lifting devices	Mobile lift	1 (0/1)	1	Used as a hospital device when function was at its worst, and no longer used once function improved; also reported to occupy too much space [H, R, S]
3 24 13 Remote control aids	Personal environment control software	1 (1/0)	1	Could not be used because it did not recognize the user’s speech well [F]
3 24 24 Positioning aids	Non-slip pads	5 (5/0)	2	Worn out, so discarded; felt at risk of tripping over the pad [D, E]

Reason codes: R, functional improvement recorded; H, use confined to the hospitalization stage or to facility equipment; M, compensation with the unaffected side; P, replaced by another device; C, cost; S, size, bulk, or lack of space at home; U, continuing need not met after discharge; F, poor fit or discomfort with the body or with the user’s abilities; D, wear, breakage, or poor durability; E, mismatch with the home or public environment; A, administrative burden or loss of eligibility; L, device lost. Codes list the reasons recorded for a device; they do not indicate how many participants gave each reason, which the summary records do not allow. Device classification codes follow the Korean assistive device classification system [46]. Reasons are summarized from the interviewers’ field notes and are not verbatim transcriptions; they consolidate the reasons recorded in the device-by-device history with those recorded in the summary tabulation, so a device may carry more reasons than it has nonusers. Acquisition is shown as (purchased/rented). Where the summary tabulation did not record a split, the number of rentals follows the device-by-device history and Table 5, and acquisitions not recorded as rentals are shown as purchases. Rows in which the number of nonusers is zero are retained to show the scale of use for that device; the reasons recorded against those rows describe replacement of a device by another of the same function rather than nonuse, as defined in Section 1.2. Where a device has more nonuse reasons listed than nonusers, the additional entries were recorded during the device history for that device type and include reasons for replacement or for nonpurchase.

**Table 7:** Reasons recorded for the devices on which the interpretation of stage-limited nonuse rests, and whether functional improvement can be isolated from other reasons.

Assistive Device	Stopped/Users	Reasons Recorded (Codes)	Can Functional Improvement Be Isolated?	Interpretation Supported by the Records
Standing frame and standing support	9/9	Use confined to inpatient rehabilitation (H); gradual functional improvement (R); too expensive (C) and too bulky (S) to consider for the home; still needed after discharge, one participant (U); leg pain (F)	No: R was recorded together with C and S, and U was recorded for one participant	End of facility-based access as much as recovery
Walker	5/5	No longer matched functional level after the early gait-training period (R)	Yes: R was the only reason recorded for this device	Recovery-related discontinuation
Standard manual wheelchair	8/10	Functional improvement (R); deliberate nonuse to practice walking (R); used in hospital and discarded after discharge (H); little used after a powered wheelchair was bought (P); discarded when tires deteriorated (D); kept in storage without use	No: reasons cannot be assigned one-to-one to the eight participants	Recovery and replacement by another device, not separable
Arm, trunk, and leg exercise device	5/10	Functional improvement (R); rented and returned because the home was small (S); too large and too expensive to purchase (S, C); facility-only use (H)	No	Recovery, space, and cost, not separable
Ankle-foot orthosis	4/6	Uncomfortable after self-modification (F); stretched and discarded (D); functional improvement (R); switched to a cane (P)	No: R was one of four distinct reasons	Chiefly fit and durability; recovery a minor contributor

Codes as in Table 6. Functional improvement is treated as isolable only where it was the sole reason recorded for the device. Where more than one reason was recorded, the summary records do not assign reasons to individual participants, so the number of discontinuations due to recovery alone cannot be counted.

**Table 8:** Reasons for nonpurchase of required assistive devices (*n* = 10).

Reason for Nonpurchase	No. of Cases	Relevant Assistive Devices (No. of Cases)
Desired product reported as not available on the market	6	Exercise, strength, and balance training equipment (3); walking-aid accessories (2); clothing and footwear (1)
High cost combined with insufficient subsidies	4	Exercise, strength, and balance training equipment (3); clothing and footwear (1)
Incompatibility with the user’s environment	4	Exercise, strength, and balance training equipment (3); clothing and footwear (1)
Difficulty finding information on suitable assistive devices	3	Pressure-relief devices (1); fixation aids (1); cooking aids (1)
Using the device makes the person feel bad (psychological reluctance)	2	Arm orthoses (2)

**Table 9:** Facilitators and barriers to assistive device utilization by the people-centered assistive technology (5P) framework.

Domain	Facilitators	Barriers
People (users)	High psychological acceptance of devices and strong information literacy; financial capacity; active social participation; burden-relieving effect for caregivers	Low psychological acceptance of devices and weak information literacy; lack of financial capacity; continuous need for custom devices due to changes in body shape and function
Policy	Privately or publicly funded devices; addition of new device items to funding programs; use of vouchers	Desired products not included in funded item lists; needed products not commercialized, reported to involve complex certification procedures; high copayment rates for high-function devices; insufficient support for custom-made devices
Products	Good compensation of bodily function; high robustness, safety, and aesthetics; form well matched to the environment (size, shape, etc.); convenient operation; high specification (for powered products)	Need for custom fabrication; poor fit with the body (continuously changing physical function and form); form mismatched to the environment (size, shape, etc.); frequent breakdowns and difficulty with after-sales service; poor wearing comfort and aesthetics
Personnel (including suppliers)	Recommendation of devices appropriate to the user; rapid awareness of device support policies and items	Device recommendations that do not consider the user’s individual environment (limited expertise on devices needed in specific settings)
Provision (service delivery)	Device rental through centers and institutions; referrals through associations (secondhand, donated, etc.)	Rental period limits; shortage of channels for obtaining device information

**Table 10:** Assistive device use, replacement, and unmet purchase across disability types in the research series.

Disability Group	Subgroup (*n*; Mean Age, Years; Mean Duration of Disability, Years)	No. of Device Types Used	No. of Device Replacements	No. of Required (Unpurchased) Devices
Physical disability	Spinal cord injury (*n* = 15; 51.5 ± 12.9; 18.4 ± 8.3)	12.5 (±4.0)	8.4 (±4.4)	3.5 (±2.2)
	Muscular dystrophy (*n* = 15; 43.2 ± 12.7; 29.7 ± 6.0)	10.5 (±3.5)	3.8 (±1.7)	3.0 (±1.5)
Brain lesion disability	Cerebral palsy (*n* = 15; 25.7 ± 9.1; 25.1 ± 8.5)	11.0 (±4.4)	13.7 (±8.9)	1.7 (±1.4)
	Stroke, this study (*n* = 10; 65.7 ± 5.0; 13.9 ± 4.6)	11.2 (±3.3)	9.8 (±7.2)	1.3 (±1.3)

Values are mean (±SD). Counts are lifetime totals since onset and are not adjusted for group size, age, or duration of disability; the groups are therefore not directly comparable. Data for spinal cord injury, muscular dystrophy, and cerebral palsy are from the earlier reports in this research series [17,48].

## Data Availability

The qualitative data generated in this study, comprising interviewer field notes and completed device checklists, are not available. Participants consented to participation on the understanding that their records would be used only by the research team, and the approved study protocol (NRC-2023-01-009) does not provide for the sharing of individual-level records. The records also combine home-setting observations with detailed device and health histories from a small, single-region sample and cannot be reliably de-identified. All data supporting the reported findings are presented in aggregated form in the article and its tables. The interview guide and the stroke-specific assistive device checklist used in this study are described in reference [14]. The 2020 National Survey on Persons with Disabilities is third-party data obtained from the Korea Institute for Health and Social Affairs and is available from that institution subject to its data access procedures.

## Abbreviations

The following abbreviations are used in this manuscript:

5P	People, Policy, Products, Personnel, Provision (WHO people-centered assistive technology framework)
ADL	Activities of daily living
BBS	Berg Balance Scale
COREQ	Consolidated Criteria for Reporting Qualitative Research
FAC	Functional Ambulation Category
GREAT	Global Research, Innovation, and Education on Assistive Technology
HAAT	Human–Activity–Assistive Technology
ICF	International Classification of Functioning, Disability and Health
IRB	Institutional Review Board
K-MBI	Korean version of the Modified Barthel Index
K-MMSE	Korean version of the Mini-Mental State Examination
MAS	Modified Ashworth Scale
MFT	Manual Function Test
WHO	World Health Organization

## Appendix A

**Table A1:** Anonymized examples of the path from summarized field-note entries to initial codes and reported categories.

Summarized Field-Note Entry (Device)	Initial Codes	Category (Reason Code)	Reported in
Standing frame: used only while receiving treatment in the rehabilitation hospital; too expensive and too bulky to consider for the home; function gradually improved, so no longer needed.	Facility-only use; cost; bulk; functional improvement	Stage-limited device, co-occurring reasons (H, C, S, R)	Table 6 and Table 7; Section 3.2
Standing frame: felt it was still needed after discharge but did not obtain one.	Continuing need; no access after discharge	Stage-limited device with unmet need (U)	Table 6 and Table 7; Section 3.2
Walker: used for gait practice early on, when function was poorer; later did not suit the user’s functional level.	Early gait training; mismatch with improved function	Recovery-related discontinuation (R)	Table 6 and Table 7; Section 3.2
Ankle-foot orthosis: the ankle part was bothersome when walking, so it was cut; afterwards it was uncomfortable and was discarded.	Discomfort; self-modification; disposal	Body-fit mismatch, abandonment (F)	Table 6 and Table 7; Section 3.2
Manual wheelchair: after discharge, deliberately not used in order to practice walking.	Intentional nonuse; gait practice	Recovery-related discontinuation (R)	Table 6 and Table 7; Section 3.2
Shower/bath chair: the bathroom was small and the chair made it narrower; discarded.	Lack of space; disposal	Environmental incompatibility (E)	Table 6; Section 3.2
Fixed grab rail: worn; reinstalling it after moving seemed bothersome; applying for support is burdensome.	Wear; relocation; administrative burden	Durability and administrative burden (D, A)	Table 6; Section 3.2
Arm orthosis, needed but not purchased: using it makes the person feel bad.	Negative feeling toward device	Psychological reluctance (nonpurchase)	Table 8; Section 3.3
Exercise device, needed but not purchased: wanted a compact home device that massages the hand or rotates the ankle or arm; could not find one on the market.	Desired product; not found; compact home use	Product not available on the market (nonpurchase)	Table 8; Section 3.3

Entries are English summaries of the interviewers’ Korean field notes and are not verbatim quotations; they are shown without participant numbers to prevent identification. The field notes did not distinguish whether an entry was stated by the participant or the caregiver; differences were reconciled during the session (Section 4.7). Reason codes are as defined in Table 6.

## Appendix B

**Table A2:** Completed 32-item Consolidated Criteria for Reporting Qualitative Research (COREQ) checklist [47]. Section numbers refer to this manuscript. Items 19, 22, 23, and 29 are marked not applicable because the interviews were not audio-recorded and no transcripts were produced.

No.	COREQ Item	Reported in
Domain 1: Research team and reflexivity		
1	Interviewer or facilitator: which author conducted the interview?	Section 2.3. Two authors (E.R., S.-Y.L.) conducted all interviews.
2	Credentials: what were the researcher’s credentials?	Section 2.3. Both hold a master’s degree, one in service-convergence design and one in human factors engineering.
3	Occupation: what was their occupation at the time of the study?	Section 2.3. Researchers at the National Rehabilitation Center.
4	Gender: was the researcher male or female?	Section 2.3. Both interviewers were female.
5	Experience and training: what experience or training did the researcher have?	Section 2.3. Both had completed interviewer training before data collection.
6	Relationship established prior to study commencement?	Section 2.3. Neither interviewer had any relationship with the participants before the study.
7	Participant knowledge of the interviewer: what did participants know about the researcher?	Section 2.3. The purpose, methods, and content of the study were fully explained before the interviews and written informed consent was obtained.
8	Interviewer characteristics: what characteristics were reported about the interviewer?	Section 2.3. Disciplinary backgrounds are reported, with a statement that these oriented the inquiry toward product usability and device–environment fit.
Domain 2: Study design		
9	Methodological orientation and theory	Section 2.1 and Section 2.4. Qualitative descriptive design with inductive qualitative content analysis; instruments structured by the HAAT model; facilitators and barriers classified with the WHO 5P framework.
10	Sampling: how were participants selected?	Section 2.2. Purposive sampling from the outpatient clinic of the National Rehabilitation Center and through staff referral.
11	Method of approach: how were participants approached?	Section 2.2. Direct approach at the outpatient clinic and staff referral.
12	Sample size: how many participants were in the study?	Section 2.2 and Table 2. Ten participants; sample size adequacy was appraised using the concept of information power.
13	Non-participation: how many people refused to participate or dropped out?	Section 2.2. All individuals approached agreed to participate and no participant withdrew.
14	Setting of data collection: where were the data collected?	Section 2.3. All interviews were conducted in the participant’s own home.
15	Presence of non-participants: was anyone else present?	Section 2.2. Caregivers participated jointly where applicable, and information on devices used by caregivers was collected. Accounts are treated as joint participant–caregiver reports (Section 2.4 and Section 4.7).
16	Description of sample: what are the important characteristics of the sample?	Table 2 and Table 3. Sex, age, diagnosis, age at onset, duration of disability, and functional assessment scores are reported for all participants.
Domain 3: Data collection		
17	Interview guide: were questions, prompts, or guides provided?	Section 2.3. The guide was organized into four HAAT domains; its development and content are described. The full instrument is available from the corresponding author.
18	Repeat interviews: were repeat interviews carried out?	Section 2.3. Each participant was interviewed twice, one week apart.
19	Audio or visual recording: did the research use recording?	Not applicable. Section 2.3 states that the interviews were not audio-recorded by design and that both interviewers documented participant responses and observations in detailed field notes during and immediately after each session.
20	Field notes: were field notes made during or after the interview?	Section 2.3. Detailed field notes were made by both interviewers during and immediately after each session and served as the analytic material.
21	Duration: what was the duration of the interviews?	Section 2.3. One to one and a half hours per session.
22	Data saturation: was data saturation discussed?	Not claimed. Sample size adequacy was appraised through information power (Section 2.2); saturation was not formally assessed, and this is stated in Section 4.8.
23	Transcripts returned: were transcripts returned to participants for comment?	Not applicable, as no transcripts were produced. Interpretations were instead returned to participants for confirmation at the second interview (Section 2.4).
Domain 4: Analysis and findings		
24	Number of data coders: how many data coders coded the data?	Section 2.4. Two researchers coded the field notes independently.
25	Description of the coding tree	Section 2.4. Categories were derived inductively using Atlas.ti; the resulting categories of nonuse reasons and of facilitators and barriers are presented in Table 6, Table 7, Table 8 and Table 9, with reason codes in Table 6 and Table 7; examples of the path from field notes to codes and categories are given in Table A2.
26	Derivation of themes: were themes identified in advance or derived from the data?	Section 2.4. Categories were derived inductively from the field notes; the 5P framework was applied only to organize the facilitator and barrier categories after derivation.
27	Software: what software was used to manage the data?	Section 2.4. Atlas.ti for coding and a spreadsheet for parallel device counts; IBM SPSS Statistics version 28 for the secondary survey analysis (Section 2.5).
28	Participant checking: did participants provide feedback on the findings?	Section 2.4. Coding discrepancies that remained uncertain were resolved by querying the participant directly at the second interview, providing a form of respondent validation.
29	Quotations presented: were participant quotations presented?	Not applicable. Because no recordings were made, no verbatim quotations are reported (Section 2.3). Reasons in Table 6 and Table 8 are summaries from field notes, as stated in the table footnotes, and individual cases are identified by participant number (P1 to P10). Anonymized examples of summarized entries are given in Table A2.
30	Data and findings consistent: was there consistency between the data presented and the findings?	Section 3.1, Section 3.2, Section 3.3, Section 3.4, Section 3.5 and Section 3.6. Device-level counts and reasons are presented in Table 4, Table 5, Table 6, Table 7, Table 8 and Table 9, and every claim in the Results is traceable to a specific table row.
31	Clarity of major themes: were major themes clearly presented?	Section 3.2, Section 4.1, Section 4.2 and Section 4.3. Two clusters of nonuse are presented, discontinuation of stage-limited devices and mismatch of body-worn devices, and are distinguished terminologically in Section 1.2.
32	Clarity of minor themes: is there a description of diverse cases or minor themes?	Section 3.2. Minor patterns are reported, including environmental incompatibility, durability and quality problems, administrative burden, and mismatch with public infrastructure; a case in which a device was still needed after discharge is also reported.
